# Key Metabolite Differences Between Korean Pine (*Pinus koraiensis*) Seeds With Primary Physiological Dormancy and No-Dormancy

**DOI:** 10.3389/fpls.2021.767108

**Published:** 2021-11-22

**Authors:** Yuan Song, Xiaoye Gao, Yunjie Wu

**Affiliations:** ^1^College of Eco-Environmental Engineering, Guizhou Minzu University, Guiyang, China; ^2^Karst Environmental Geological Hazard Prevention Laboratory of Guizhou Minzu University, Guiyang, China

**Keywords:** Korean pine, dormancy, germination, metabolomics, embryo, megagametophyte

## Abstract

*Pinus Koraiensis* seeds have physiological dormancy. Cold stratification releases seed dormancy. The changes in metabolite profiles of dormant seeds and cold stratified seeds during shorter incubation time in a favorable condition for seed germination have been studied. However, a more-long-term detection of the changes in metabolites in dormant seeds can identify the real metabolic pathways responsible for dormancy. Metabolite composition was investigated in embryo and megagametophyte of primary physiological dormant seeds (DS) of *P. Koraiensis* collected at 0, 1, 2, 4, and 6 weeks of incubation and of non-primary physiological dormant seeds (NDS) sampled at 0 and 1 week of incubation, seed coat rupture stage, and radicle protrusion stage. Embryos contained higher levels of most metabolites than megagametophyte. Strong accumulation of most metabolites in DS occurred at 1 and 4 weeks of incubation. A larger reduction in the relative levels of most phosphorylated sugars and amino acids in NDS was found between 1-week-incubation and seed coat rupture stage. The relative levels of metabolites involved in carbohydrate metabolism, especially the pentose phosphate pathway (PPP) and tricarboxylic acid (TCA) cycle, were higher in the embryos of 4-week-incubated DS, but the relative contents of intermediate metabolites of most amino acid metabolism were lower compared to 1-week-incubated NDS. We suggested that the disturbed carbohydrate metabolism and amino acid metabolism in the embryos of DS after 4 weeks of incubation maybe related to primary dormancy. Our study provides information for a better understanding of the mechanism of seed dormancy.

## Introduction

Seed germination is a very complex physiological and biochemical process that is mainly determined by genetic factors with a substantial environmental influence (Joosen et al., 2013). A series of physiological and biochemical processes is initiated in imbibed seeds, such as repair mechanisms, transcription, translation, reactivation of metabolism, reserves mobilization, redox homeostasis regulation, and organellar reassembly (He et al., 2011; Bewley et al., 2013). Germination is driven by these biochemistry processes. The combination of high-throughput and large-scale-omics methods is used to understand the regulation of seed germination. Due to the importance of agriculture and the availability of genome sequence data, the genomic, transcriptomic, and proteomic studies of seed germination have largely focused on model species, such as *Arabidopsis thaliana* (Nakabayashi et al., 2005; Holdsworth et al., 2008a,b; Narsai et al., 2017a), rice (*Oryza sativa*) (Narsai et al., 2017b), maize (*Zea mays*) (Sekhon et al., 2013), barley (*Hordeum vulgare*) (Lin et al., 2014), and wheat (*Triticum aestivum*) (Yu et al., 2014). These studies revealed that metabolism-related genes are also highly expressed during germination (Nakabayashi et al., 2005). Metabolism-related proteins are the major groups detected in germinating rice seeds (He et al., 2011; Sano et al., 2012). The central carbon metabolism pathways provide energy and building blocks for various metabolic activities (Rosental et al., 2014). Glycolysis, fermentation, the tricarboxylic acid (TCA) cycle, and the oxidative pentose phosphate pathway (PPP) are activated to provide energy for seed germination (Yang et al., 2007; He et al., 2011). Despite recent advances in omics studies of seed germination, few experiments focus on the mechanisms involved in seed dormancy in woody plants from a metabolomic point of view.

Seed dormancy refers to an intact viable seed that is not able to germinate in a specified period of time under favorable conditions (Baskin and Baskin, 2014). Seed dormancy can be divided into primary dormancy and secondary dormancy on the basis of the timing of the acquisition of dormancy (Bewley et al., 2013). Primary dormancy develops during seed maturation (Bewley et al., 2013). Secondary dormancy is induced by various external environmental factors following seeds dispersal from the mother plant (Bewley et al., 2013). The types of seed dormancy also include physiological dormancy (physiological inhibiting factors in the embryo), morphological dormancy (the embryo is undifferentiated or underdeveloped), physical dormancy (the seed coat is impermeable to water), morphophysiological dormancy (a combination of morphological dormancy and physiological dormancy), and combinational dormancy (a combination of physical dormancy and physiological dormancy) (Baskin and Baskin, 2014). Non-dormant is defined as the capacity of seeds to germinate over the widest range of environmental factors (Baskin and Baskin, 2014). Cold stratification or after-ripening is generally used to break seed dormancy.

Several studies have investigated proteomic, transcriptomic, or metabolic changes during either cold stratification or after-ripening in *Prunus campanulata* (Lee et al., 2006), *A. thaliana* (Angelovici et al., 2011; Arc et al., 2012), *Acer pseudoplatanus* (Pawłowski and Staszak, 2016), *Gnetum parvifolium* (Chang et al., 2018), and *Arachis hypogaea* (Xu et al., 2020). The mechanism of seed dormancy break is thus well studied and understood. Seed dormancy and germination mechanisms are generally studied by comparing dormant and non-dormant seeds. However, the following points should be noted in this study. First, it is the metabolic activities occurring before radical protrusion that are responsible for seed germination (Bewley et al., 2013). Acquiring a comprehensive knowledge of metabolic changes at this stage is thus necessary. Second, only when seeds are incubated at favorable conditions for a period of time, we can determine seed is dormant or not. It is also worth considering metabolic changes during the incubation of dormant seeds. Third, water imbibition itself can trigger changes in gene expression or the entire metabolism regardless of the depth of seed dormancy (Preston et al., 2009; Yazdanpanah et al., 2017; Xia et al., 2018b). Thus, both dormant and non-dormant seeds should be maintained at an imbibed state. Comparative transcriptomic or metabolomic studies investigation has been performed on imbibed dormant and after-ripened seeds to reveal mechanisms controlling seed germination in *A. thaliana* (Cadman et al., 2006; Yazdanpanah et al., 2017), wheat (Gao et al., 2012), and sunflower (*Helianthus annuus*; Xia et al., 2018b). However, for seeds that required cold stratification to release seed dormancy, the metabolic changes in dormant seeds and cold stratified seeds under favorable conditions have not been determined. Fait et al. (2006) have shown that the relative levels of TCA-cycle intermediates were increased during stratification and were further enhanced during germination *sensu stricto of A. thaliana* seeds. However, how the metabolites in dormant seeds change under favorable conditions is still unknown.

Different seed parts, such as embryo and endosperm, have specific functions during seed germination (Baskin and Baskin, 2004; Lee et al., 2006; Xu et al., 2016). The biochemical changes occurring in different seed tissues were not investigated in some species, such as rice, wheat, and poplar, due to a limitation of sample separation technique (Ferrari et al., 2009; Gao et al., 2012; Qu et al., 2019). The real differential metabolic activities between dormant and non-dormant seeds are possibly ignored given the use of mixed tissues over the experimental period. This requires in future work distinguishing properly the roles of embryo from the endosperm in either dormancy release and subsequent germination or dormancy maintenance.

Korean pine (*Pinus Koraiensis*) is a dominant species in mixed-broadleaved Korean pine forests in Northeast China. Korean pine seeds have primary physiological dormancy. Previous metabolomic study of Korean pine seeds revealed that the relative levels of metabolites related to glycolysis pathway and TCA cycle in the embryos of non-primary physiological dormant seeds (NDS) substantially were decreased after 11 days of incubation in favorable conditions. In contrast, this metabolic change was not observed in primary physiological dormant seeds (DS). They thus hypothesize that primary physiological dormancy maybe closely associated with the glycolysis pathway and TCA cycle (Song and Zhu, 2019). Whether those metabolites related to glycolysis pathway and TCA cycle in DS also maintain high levels as the increasing incubation period is however still unclear. It is possible that other strong metabolic changes occurring in DS are responsible for seed primary physiological dormancy. Furthermore, the levels of abscisic acid (ABA) in both DS and NDS did not change significantly after 11 days of incubation (Song et al., 2020). Thus, 11 days is too short to detect the metabolic changes associated with Korean pine seed primary physiological dormancy. It is necessary to prolong incubation time. In addition, the metabolic changes occurring in megagametophyte were also not determined, making it impossible to reveal metabolic differences between DS and NDS. Thus, the objective of this study was to compare metabolomic profiles of embryos and megagametophytes from dry DS and NDS during a longer period of incubation under favorable conditions.

## Materials and Methods

### Seed Collection

In late September 2018, fresh Korean pine seeds were collected from at least 50 trees from virgin mixed-broadleaved Korean pine forest in Wuying Fenglin National Nature Reserves in Northeastern China (128°58′–129°15′E, 48°02′–48°12′N). Seed water content was only about 10% (dry weight basis). These fresh dry seeds were stored at −20°C until dormancy status determination.

### Seed Burial Experiment

On October 20, 2018, approximately 500 Korean pine seeds were mixed with soil. The mixture of seed and soil was placed in a 40 cm × 40 cm fine-mesh nylon bag. The nylon bag was then put into a metal mesh box to avoid predation by animals. Finally, the metal bags were buried between litterfall and soil for approximately 10 cm depth in virgin mixed-broadleaved Korean pine forest. The primary physiological dormancy of Korean pine seed would be released under the effect of lower late autumn and winter temperatures in natural conditions (Song and Zhu, 2016). In late May 2019, seed bags were retrieved from virgin mixed-broadleaved Korean pine forest. The seeds in the nylon bag were soaked in deionized water and those sinking seeds with testa intactness were collected and stored at 4°C for less than 1 week until dormancy status determination.

### Determination of Seed Dormancy Status

The dormancy status of seeds collected in September 2018 and May 2019 was determined at four incubation temperature regimes (10/5, 20/10, 25/15, and 30/20°C). The detailed germination test steps could be found in Song and Gao (2021). Seeds collected in September 2018 were germinated to only 3–32% at four temperature regimes, indicating that these seeds are primary physiological dormant. Seeds collected in May 2019 can be germinated to 27–90% at four temperature regimes, indicating that these seeds are non-primary physiological dormant (Song and Gao, 2021).

### Incubation Experiment

Both DS and NDS were incubated in a growth incubator under a temperature fluctuation regime of 25°C for 14 h and 15°C for 10 h. Light, 200 μmol of photons m^–2^ s^–1^ was provided by cool white fluorescent tubes during high temperature (14 h), and darkness was set during low temperature (10 h). DS were incubated for 6 weeks and sampled on 0, 1, 2, 4, and 6 weeks after incubation; abbreviated as DS-0, DS-1, DS-2, DS-4, and DS-6, respectively. NDS were incubated and sampled at 0 week of incubation, 1 week of incubation, seed coat rupture stage, and radicle protrusion stage; abbreviated as NDS-0, NDS-1W, NDS-SCR, and NDS-RP, respectively.

Five replicates of 20 DS or NDS for each time point were placed in 10-cm Petri dishes with five layers of 11-cm filter paper (Jiaojie, Fushun, China) moistened with 10 ml deionized water. Petri dishes were then covered with parafilm to reduce water loss.

### Seed Samples Pretreatment

The relative levels of metabolites in the embryos of five DS samples, the megagametophytes of five DS samples, the embryos of four NDS samples, and the megagametophytes of four NDS samples were all used for non-targeted metabolome analysis. The excised embryos and megagametophytes were immediately frozen in liquid nitrogen, separately and subsequently stored at −80°C. These embryos and megagametophytes samples were ground into powder under the protection of liquid nitrogen. Eighty milligrams of the embryo and megagametophyte powders were placed separately inside a 1.5-ml Eppendorf tube (Eppendorf, Hamburg, Germany). One milliliter of precooled extraction solution [methanol/acetonitrile/ddH_2_O (2:2:1, v/v/v)] was then added (Pi et al., 2018). The mixture of powder and extraction solution was then subjected for following treatments, vortex for 60 s, low-temperature ultrasonic for 30 min (twice), stands at −20°C for 1 h to precipitate protein, and centrifugation for 20 min (14,000 relative centrifugal force, 4°C) (Pi et al., 2018). After that, the supernatant was lyophilized and stored at −80°C until used for further metabolomics analysis (Pi et al., 2018).

### Liquid Chromatography-Mass Spectrometry Analysis

The UHPLC/MC system consisted of an Agilent 1290 Infinity LC (Agilent Technologies, Santa Clara, CA, United States) connected to a TripleTOF 6600 mass spectrometer (AB SCIEX, Framingham, MA, United States). Chromatographic analyses were performed using a hydrop interaction liquid chromatography (HILIC) column (ACQUITY UPLC BEH Amide 2.1 mm × 100 mm column, internal diameter 1.7 μm, Waters, Ireland) maintained at 25°C, and the autosampler tray temperature was maintained at 4°C. Mobile phase A was water with 25 mM ammonium acetate and 25 mM ammonia; mobile phase B was acetonitrile. The gradient conditions were 0–1 min, 95% B; 1–14 min, from 95 to 65% B; 14–16 min, from 65 to 40% B; 16–18 min, 40% B; 18–18.1 min, from 40 to 95% B, and 18.1–23 min, 95% B. The flow rate was 0.3 ml min^–1^, and the injection volume was 2 μl. For both modes of operation, the mass spectrometric parameters were as in Zhou et al. (2019).

### Statistical Analysis

Raw data (wiff. scan files) were converted to mzXML format using ProteoWizard. Then, peak alignment, retention time correction, and peak area extraction were conducted with the XCMS program.

Metabolite structure identification was performed with accurate mass number matching (<25 ppm) and secondary spectrum matching. The ion peaks with greater missing values (>50%) were removed. Metabolites were searched in a laboratory self-built database. Statistical analyses were conducted with MetaboAnalyst^1^.

Principal component analysis (PCA) was used to investigate the metabolic changes in both embryos and megagametophytes during incubation of DS or NDS. For PCA performed on the relative contents of metabolites in the embryos and megagametophytes of five types of DS, the metabolite data were log transformed (generalized logarithm transformation) and auto scaled (mean-centered and divided by the standard deviation of each variable) for normalization. To gain a better normalization result, Pareto scaling (mean-centered and divided by the square root of the standard deviation of each variable) was used for the metabolite data obtained from the embryos and megagametophytes of four types of NDS. We also used PCA to analyze the alterations in metabolic profiles of the embryos (or megagametophytes) of both DS and NDS. The metabolite data of the embryos of DS and NDS were log transformed and auto scaled for normalization and then used for PCA. The metabolite data of the megagametophytes of DS and NDS were also subjected to PCA after auto scaling for normalization.

Partial least squares-discriminant analysis (PLS-DA) was conducted over 20 pairs of samples (where E is embry and M is megagametophyte) (DS-0-E vs. DS-1-E, DS-1-E vs. DS-2-E, DS-2-E vs. DS-4-E, DS-4-E vs. DS-6-E, DS-1-E vs. DS-4-E, DS-1-E vs. DS-6-E, DS-0-M vs. DS-1-M, DS-1-M vs. DS-2-M, DS-2-M vs. DS-4-M, DS-4-M vs. DS-6-M, DS-1-M vs. DS-4-M, DS-1-M vs. DS-6-M, NDS-0-E vs. NDS-1-E, NDS-1-E vs. NDS-SCR-E, NDS-SCR-E vs. NDS-RP-E, NDS-0-M vs. NDS-1-M, NDS-1-M vs. NDS-SCR-M, NDS-SCR-M vs. NDS-RP-M, DS-4-E vs. NDS-1-E, DS-4-M vs. NDS-1-M, DS-4-E vs. NDS-SCR-E, and DS-4-M vs. NDS-SCR-M) to determine whose metabolites contribute significantly to the separation of two samples. Those metabolites with variable importance in the projection (VIP) >1 and significant changes (*P* < 0.05) in relative contents were differentially expressed between the two samples. The metabolite data were also subjected to a combined normalization treatment of log transformation and auto scaling (or Pareto scaling alone) before performing PLS-DA.

Fold change (FC) in the relative contents of the metabolites (VIP > 1 and *P* < 0.05) in the embryos and megagametophytes of DS and NDS, separately, was determined between different incubation time points (0 vs. 1, 1 vs. 2, 2 vs. 4, 4 vs. 6, 1 vs. 4, 1 vs. 6, 1 vs. SCR, and SCR vs. RP), between different types of seeds (NDS-1 vs. DS-4 and NDS-SCR vs. DS-4), between different seed parts (embryo vs. megagametophyte). FC was calculated as the ratio of the former term to latter term and log_2_ transformed. Log_2_(FC) value greater than zero indicates a fold decrease in the relative contents of metabolites. If log_2_(FC) value is less than zero, suggesting a fold increase in the relative contents of metabolites. The FCs of metabolites were visualized by a heat map. Log_2_(FC) value was used to generate heat map using Excel 2019. Metabolic pathway analysis was also performed with metabolites (VIP > 1 and *P* < 0.05) between any of 22 pairs of samples using the MetaboAnalyst web tool to determine whose metabolic pathways are significantly changed. The relative parameters were set using the same method as that used by Song and Zhu (2019).

## Results

### Principal Component Analysis of the Relative Contents of Metabolites in Primary Physiological Dormant Seeds

**FIGURE 1:**
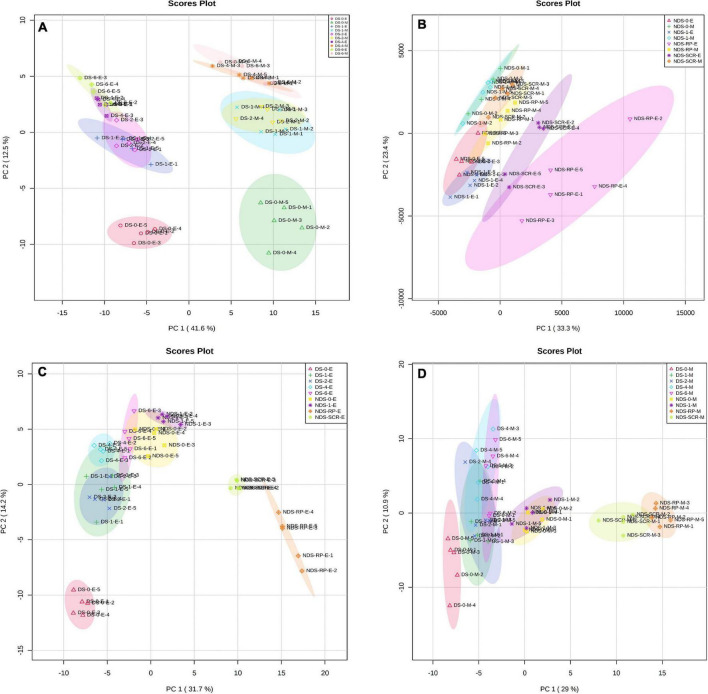
Principal components analysis (PCA) of metabolite profiles in **(A)** the embryos (E) and megagametophytes (M) of primary physiological dormant seeds (DS) sampled at 0 week of incubation (DS-0-E and DS-0-M), 1 week of incubation (DS-1-E and DS-1-M), 2 weeks of incubation (DS-2-E and DS-2-M), 4 weeks of incubation (DS-4-E and DS-4-M), and 6 weeks of incubation (DS-6-E and DS-6-M), **(B)** the embryos and megagametophytes of non-primary physiological dormant seeds (NDS) sampled at 0 week of incubation (NDS-0-E and NDS-0-M), 1 week of incubation (NDS-1-E and NDS-1-M), seed coat rupture stage (NDS-SCR-E and NDS-SCR-M), and radicle protrusion stage (NDS-RP-E and NDS-RP-M), **(C)** the embryos of DS and NDS, and **(D)** the megagametophytes of DS and NDS.

Principal component analysis was performed on DS or NDS to investigate metabolic processes at the global level. The first component of PCA clearly separated DS embryo samples from megagametophyte samples, accounting for 41.6% of the variance. DS-0-E and DS-0-M samples formed two more discrete groups on the first principal component (Figure 1A). The transition from 1W to 2W was associated with smaller differences in the relative contents of metabolites in both embryos and megagametophytes. The samples collected at 4W and 6W were also situated relatively close to one another. The metabolome of both embryo and megagametophyte in DS started to deviate at 4W on the second principal component (Figure 1A).

### Principal Component Analysis of Relative Contents of Metabolites in Non-Primary Physiological Dormant Seeds

Non-primary physiological dormant seeds embryos experienced relatively larger metabolic changes than the megagametophytes during incubation (Figure 1B). Embryos did not display distinct alterations in metabolite profile after 1 week of incubation. However, there was a large metabolic shift after seed coat rupture, indicated by the apparent separation between NDS-1-E and NDS-SCR-E samples along with the first principal component. A relatively small difference was found between NDS-1-M and NDS-SCR-M samples.

### Principal Component Analysis of the Relative Contents of Metabolites in the Embryo and Megagametophyte of Both Primary Physiological Dormant Seeds and Non-Primary Physiological Dormant Seeds

DS-4-E sample was clearly different from NDS-1-E, NDS-SCR-E, and NDS-RP-E samples on the first dimension of the PCA (Figure 1C). A similar distinction was also found between NDS-1-M, NDS-SCR-M, NDS-RP-M, and DS-4-M samples (Figure 1D).

### Fold Changes of Important Metabolites With a Variable Importance in the Projection Value >1 and *P* < 0.05 During Incubation of Primary Physiological Dormant Seeds

**FIGURE 2:**
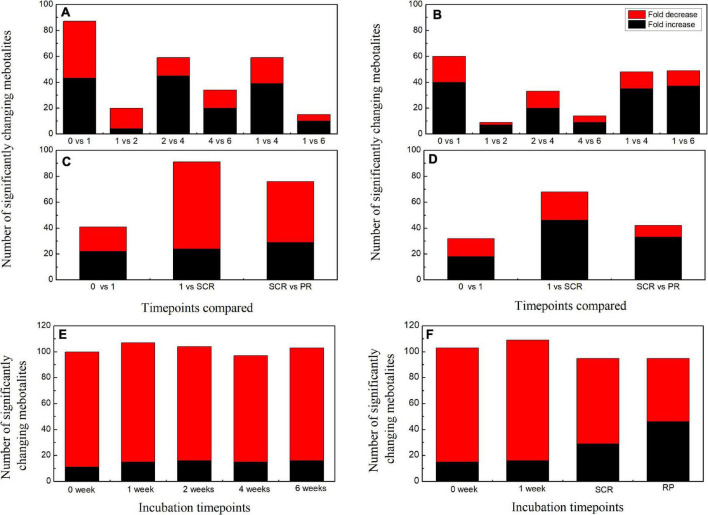
Summary of the number of significant changes in metabolites with VIP value >1 and *P* < 0.05 between different incubation time points for the embryos **(A)** and megagametophytes **(B)** of primary physiological dormant seeds (DS) and the embryos **(C)** and megagametophytes **(D)** of non-primary physiological dormant seeds (NDS). The numbers of significantly changing metabolites with VIP value >1 and *P* < 0.05 between embryo and megagametophyte at each incubation time points for DS **(E)** and NDS **(F)**. VIP, variable importance in the projection.

**FIGURE 3:**
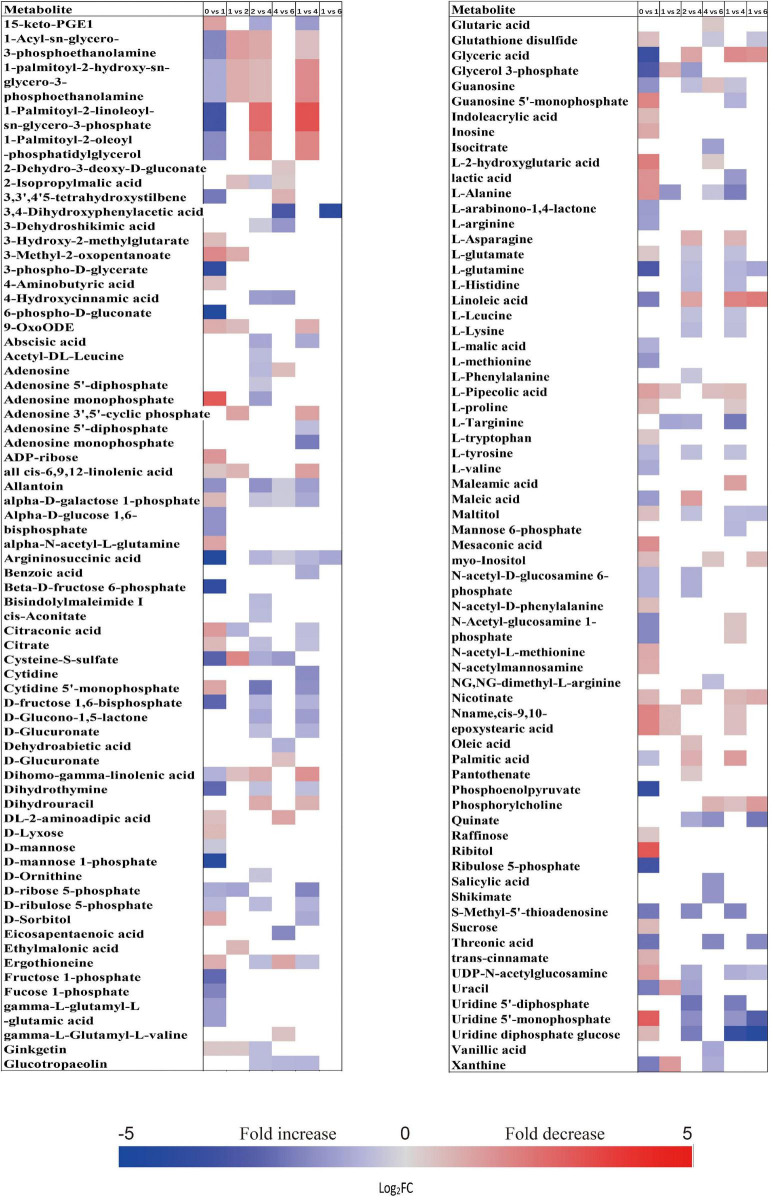
Fold change in the relative contents of important metabolites with VIP value >1 and *P* < 0.05 in the embryos of primary physiological dormant seeds between different incubation time points (0 week vs. 1 week, 1 week vs. 2 weeks, 2 weeks vs. 4 weeks, 4 weeks vs. 6 weeks, 1 week vs. 4 weeks, and 1 week vs. 6 weeks). Fold changes are represented as a heat map. White coloring indicates no significant change. VIP, variable importance in the projection.

When comparing the patterns of change in the relative contents of metabolites in embryos with VIP > 1 and *P* < 0.05 between successive time points, a large variation occurred in the first week (43 increased and 44 decreased), with few changes observed between 1 and 2 weeks (4 increased and 16 decreased), and between 4 and 6 weeks (20 increased and 14 decreased) (Figure 2A). There was a relatively higher alternation between 2 and 4 weeks (45 increased and 14 decreased) (Figure 2A). Only 15 metabolites exhibited large differences between 1 and 6 weeks (Figure 2A). Those metabolites that displayed a major increase in their relative contents after 1 week of incubation were mainly involved in major carbohydrate metabolism (Figure 3). Two TCA-cycle intermediates (citrate and *cis*-aconitate) and three phosphorylated sugars, such as D-fructose 1,6-bisphosphate, D-ribulose 5-phosphate, and alpha-D-galactose 1-phosphate, were also significantly accumulated from 2 to 4 weeks (Figure 3). The relative level of citrate, both the number and relative contents of phosphorylated sugars were also significantly higher in DS-4 than that in DS-1 (Figure 3).

**FIGURE 4:**
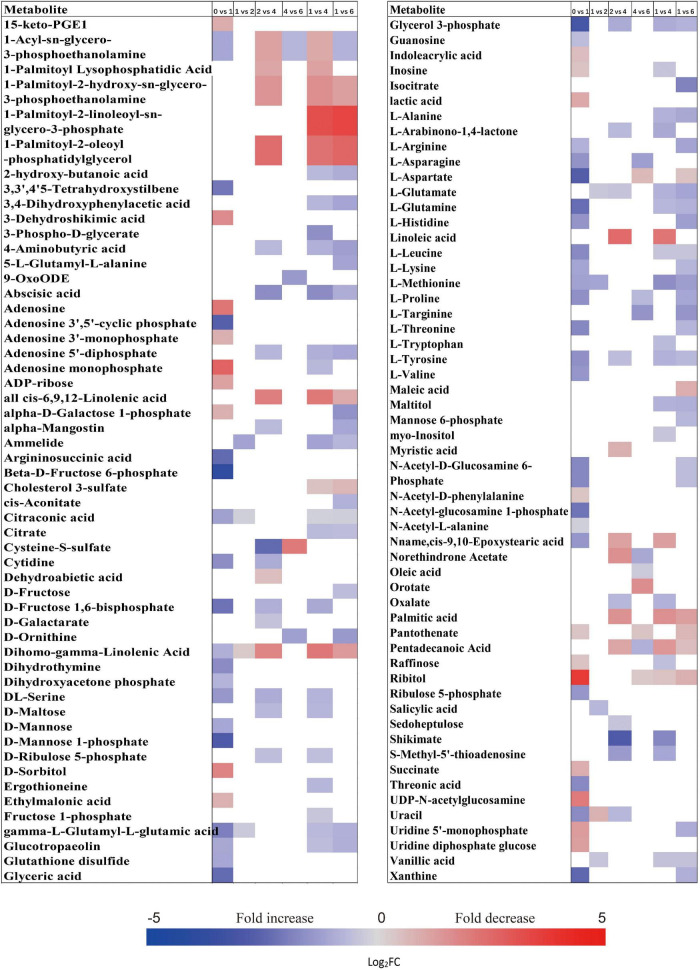
Fold change in the relative contents of important metabolites with VIP value >1 and *P* < 0.05 in the megagametophytes of primary physiological dormant seeds between different incubation time points (0 week vs. 1 week, 1 week vs. 2 weeks, 2 weeks vs. 4 weeks, 4 weeks vs. 6 weeks, 1 week vs. 4 weeks, and 1 week vs. 6 weeks). Fold changes are represented as a heat map. White coloring indicates no significant change. VIP, variable importance in the projection.

The relative amounts of most metabolites were significantly higher in embryos than that in megagametophytes at all six time points (Figure 2E). A rather similar pattern of metabolites was found in megagametophyte between successive time points, with a large metabolic change between 0 and 1 week and between 2 and 4 weeks (Figure 2B). A lesser extent of change in metabolites occurred between 1 and 2 weeks and between 4 and 6 weeks (Figure 2B). Higher numbers of amino acids and TCA-cycle intermediates were found to be significantly accumulated between 1 and 6 weeks than between 1 and 4 weeks (Figure 4).

### Fold Changes of Important Metabolites With a Variable Importance in the Projection Value >1 and *P* < 0.05 During Incubation of Non-Primary Physiological Dormant Seeds

**FIGURE 5:**
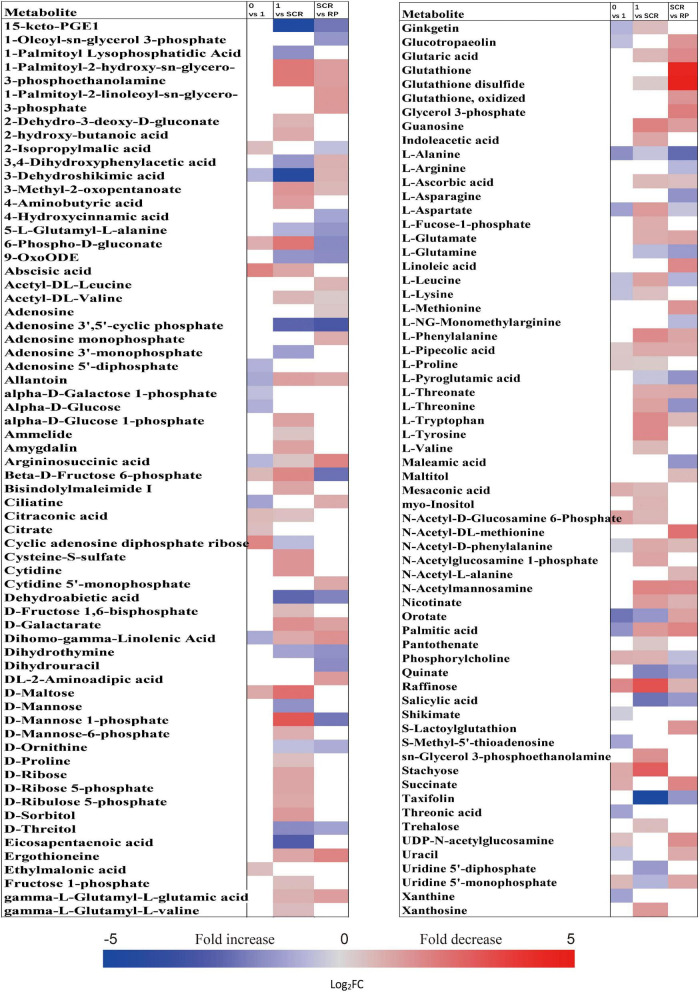
Fold change in the relative contents of important metabolites with VIP value >1 and *P* < 0.05 in the embryos of non-primary physiological dormant seeds between different incubation time points, such as 0 week vs. 1 week, 1 week vs. SCR (seed coat rupture), and SCR vs. RP (radicle protrusion). Fold changes are represented as a heat map. White coloring indicates no significant change. VIP, variable importance in the projection.

The relative contents of metabolites in the embryos of NDS were changed little over the first 1 week of incubation (16 increased and 23 decreased) (Figure 2C). The relative levels of 67 metabolites were significantly reduced in NDS-SCR compared with NDS-1 (Figure 2C). In detail, 10 phosphorylated sugars (D-mannose 1-phosphate, 6-phospho-D-gluconate, beta-D-fructose 6-phosphate, alpha-D-glucose 1-phosphate, D-ribose 5-phosphate, D-ribulose 5-phosphate, D-mannose-6-phosphate, L-fucose-1-phosphate, D-fructose 1,6-bisphosphate, and fructose 1-phosphate), two sugar alcohols (D-sorbitol and myo-inositol), two disaccharides (D-maltose and trehalose), two trisaccharide (raffinose and stachyose), and 12 amino acids displayed one to eightfold decrease (Figure 5). A number of significant changes in metabolite abundance were observed between seed coat rupture and radicle protrusion stage (76 of the 188 metabolites displayed significant changes; Figure 2C). However, the differences between the two stages were more subtle compared with the large changes in the metabolome observed from 1 week to seed coat rupture stage (Figure 2C).

**FIGURE 6:**
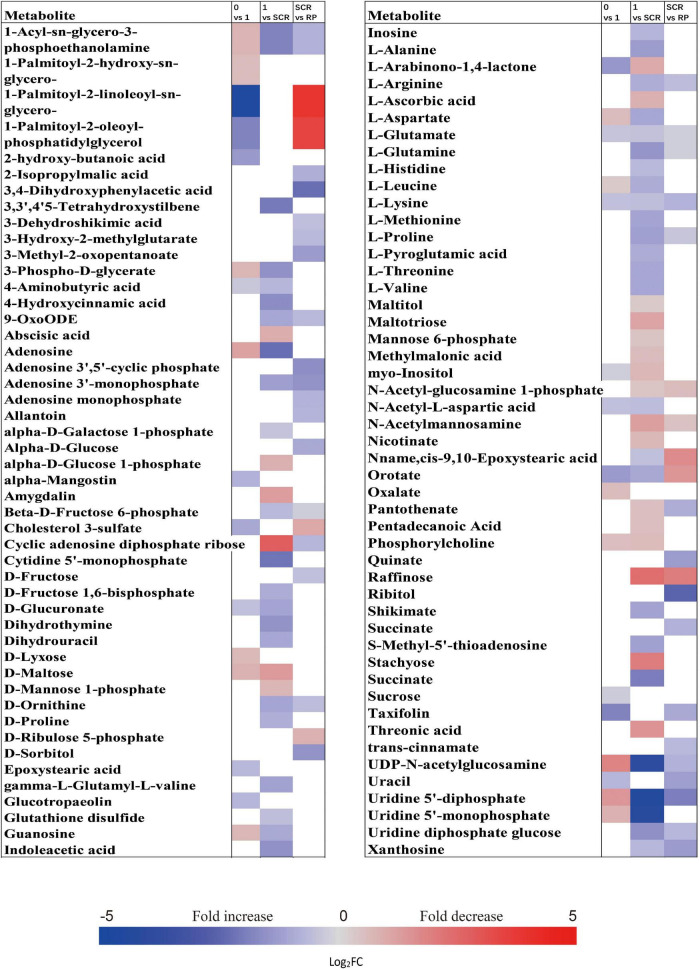
Fold change in the relative contents of important metabolites with VIP value >1 and *P* < 0.05 in the megagametophytes of non-primary physiological dormant seeds (NDS) between different incubation time points, such as 0 week vs. 1 week, 1 week vs. SCR (seed coat rupture), and SCR vs. RP (radicle protrusion). Fold changes are represented as a heat map. White coloring indicates no significant change. VIP, variable importance in the projection.

At 0 week, 1 week, and seed coat rupture stage, the embryos in NDS contained higher relative contents of most metabolites than megagametophytes (Figure 2F). After 1 week of incubation, a smaller proportion of the detected metabolites was changed in megagametophytes (Figure 2D). The trend of changes in metabolites was then followed by the largest change between 1 week and seed coat rupture stage, followed by a relatively small change between seed coat rupture and radicle protrusion stage (Figure 2D). During the period from 1 week to seed coat rupture stage, two sugar alcohols (maltitol and myo-inositol), one disaccharide (D-maltose), and three trisaccharides (raffinose, stachyose, and maltotriose) were significantly decreased one to fivefold (Figure 6). While most of the changes in amino acids were seen to occur in megagametophyte, 15 amino acids displayed 1.4- and 2.7-fold increases at the seed coat rupture stage (Figure 6).

### Fold Changes of Important Metabolites With a Variable Importance in the Projection Value >1 and *P* < 0.05 Between DS-4 and NDS-1, DS-4 and NDS-SCR

**FIGURE 7:**
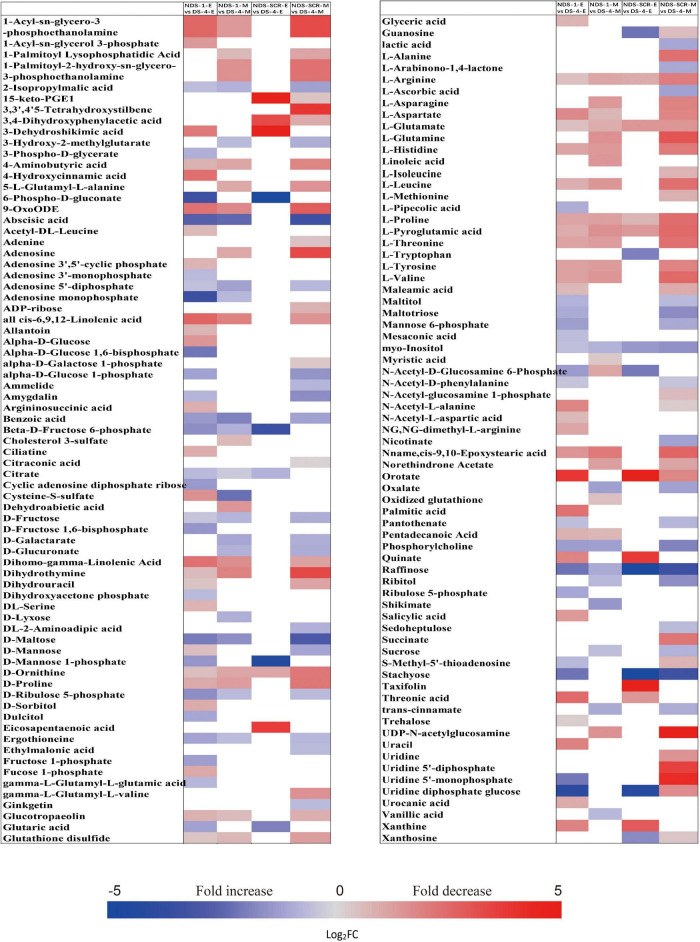
Fold change in the relative contents of important metabolites with VIP value >1 and *P* < 0.05 in the embryos (E) and megagametophytes (M) between different types of seeds, such as non-primary physiological dormant seeds (NDS) incubated for 1 week (NDS-1), primary physiological dormant seeds incubated for 4 weeks (DS-4), and NDS with seed coat rupture (NDS-SCR). Fold changes are represented as a heat map. White coloring indicates no significant change. VIP, variable importance in the projection.

The metabolic processes occurring before radicle protrusion regulate the completion of germination. In addition, a relatively large metabolic change in DS was observed after 4 weeks of incubation. Thus, we performed the FC analyses of metabolites differentially accumulated between NDS-1, NDS-SCR, and DS-4, aiming at revealing real potential metabolic processes controlling seed primary physiological dormancy maintenance. Ninety-two metabolites were found to be significantly accumulated in the embryos between NDS-1 and DS-4 (Figure 7). Of these, the relative levels of 41 metabolites were significantly higher in DS-4, such as 6-phospho-D-gluconate, ABA, raffinose, stachyose, alpha-D-glucose 1,6-bisphosphate, beta-D-fructose 6-phosphate, D-ribulose 5-phosphate, D-mannose 1-phosphate, D-fructose 1,6-bisphosphate, and citrate. The relative levels of most amino acids were higher in NDS-1 than that in DS-4. Only the relative levels of 27 metabolites were significantly different in embryos between NDS-SCR and DS-4 (Figure 7).

The relative content of ABA in megagametophyte was 5.3-fold higher in DS-4 rather than in NDS-1 (Figure 7). D-maltose, raffinose, D-lyxose, myo-inositol, D-fructose, ribitol, and sucrose also remained at high relative levels in DS-4, whereas 14 amino acids were at least twofold more highly accumulated in NDS-1 compared with DS-4. More amino acids were remained in the megagametophytes of NDS-SCR than in DS-4 (Figure 7). In contrast, raffinose, stachyose, D-maltose, maltotriose, ribitol, myo-inositol, D-fructose, sucrose, maltitol, and sedoheptulose exhibited higher relative contents in DS-4.

### The Patterns of Change in Metabolic Pathways During Incubation of Primary Physiological Dormant Seeds and Non-Primary Physiological Dormant Seeds

Most metabolites with their relative levels significantly changed during incubation of DS and NDS were mainly associated with carbohydrate metabolism, amino acid metabolism, and lipid metabolism (Tables 1, 2). For DS, a dramatic change in most metabolic pathways was observed at both 1 and 4 weeks (Table 1). There was also a strong metabolic switch in NDS from 1 week to seed coat rupture stage (Table 2).

**TABLE 1:** The altered metabolic pathways in the embryos and megagametophytes of primary physiological dormant seeds between different incubation time points.

**Metabolic pathways**		0 week vs. 1 week		1 week vs. 2 weeks		2 weeks vs. 4 weeks		4 weeks vs. 6 weeks		1 week vs. 4 weeks		1 week vs. 6 weeks	
		Em	Me	Em	Me	Em	Me	Em	Me	Em	Me	Em	Me
Carbohydrate metabolism	Pentose phosphate pathway	✓	✓	✓		✓	✓			✓	✓		
	C5-Branched dibasic acid metabolism	✓	✓	✓	✓					✓	✓		✓
	Fructose and mannose metabolism	✓	✓							✓			✓
	Amino sugar and nucleotide sugar metabolism	✓	✓			✓				✓		✓	✓
	Pentose and glucuronate interconversions	✓				✓	✓			✓	✓		
	Glycolysis/gluconeogenesis	✓											
	Pyruvate metabolism	✓											
	Glyoxylate and dicarboxylate metabolism	✓				✓		✓		✓			✓
	Starch and sucrose metabolism	✓					✓			✓			
	Galactose metabolism	✓	✓										
	Citrate cycle (TCA cycle)	✓				✓				✓	✓		✓
	Butanoate metabolism	✓					✓				✓		✓
	Carbon fixation in photosynthetic organisms	✓											
	Inositol phosphate metabolism	✓						✓			✓	✓	
Amino acid metabolism	Alanine, aspartate, and glutamate metabolism	✓	✓		✓	✓	✓		✓	✓	✓	✓	✓
	Arginine and proline metabolism	✓	✓						✓	✓			✓
	Arginine biosynthesis	✓	✓			✓				✓			✓
	Cysteine and methionine metabolism	✓	✓		✓						✓		✓
	Tryptophan metabolism	✓									✓		
	Tyrosine metabolism	✓	✓			✓	✓			✓	✓		✓
	Phenylalanine metabolism					✓							
	Glycine, serine, and threonine metabolism		✓										✓
	Phenylalanine, tyrosine, and tryptophan biosynthesis						✓				✓		
Nucleotide metabolism	Pyrimidine metabolism	✓	✓			✓				✓		✓	✓
	Purine metabolism	✓				✓				✓	✓		
Lipid metabolism	Glycerophospholipid metabolism	✓				✓	✓			✓	✓		✓
	Linoleic acid metabolism	✓				✓	✓			✓	✓	✓	
	Glycerolipid metabolism	✓				✓				✓	✓		
	Cutin, suberine, and wax biosynthesis					✓			✓				
Biosynthesis of other secondary metabolites	Isoquinoline alkaloid biosynthesis	✓	✓			✓	✓			✓	✓		✓
Biosynthesis of other secondary metabolites-unclassified						✓		✓					
Metabolism of cofactors and vitamins	Pantothenate and CoA biosynthesis		✓			✓			✓				✓
Metabolism of other amino acids	Glutathione metabolism												✓

*Em, embryo; Me, megagametophyte.*

*The symbol “✓” indicates significantly changed metabolic pathway between two time points.*

**TABLE 2:** The altered metabolic pathways in the embryos and megagametophytes of non-primary physiological dormant seeds between successive time points.

**Metabolic pathways**		0 week vs. 1 week		1 week vs. SCR		SCR vs. RP	
		Em	Me	Em	Me	Em	Me
Carbohydrate metabolism	Pentose phosphate pathway	✓		✓	✓	✓	✓
	C5-Branched dibasic acid metabolism	✓		✓			
	Fructose and mannose metabolism	✓		✓	✓	✓	✓
	Amino sugar and nucleotide sugar metabolism			✓	✓	✓	✓
	Pentose and glucuronate interconversions			✓			
	Glycolysis/gluconeogenesis						
	Pyruvate metabolism						
	Glyoxylate and dicarboxylate metabolism						
	Starch and sucrose metabolism	✓	✓	✓	✓		✓
	Galactose metabolism	✓		✓	✓		✓
	Citrate cycle (TCA cycle)	✓					
	Butanoate metabolism		✓	✓	✓		✓
	Carbon fixation in photosynthetic organisms						
	Inositol phosphate metabolism		✓	✓	✓		✓
	Ascorbate and aldarate metabolism			✓	✓	✓	✓
Amino acid metabolism	Alanine, aspartate, and glutamate metabolism	✓	✓	✓	✓	✓	✓
	Arginine and proline metabolism			✓	✓		✓
	Arginine biosynthesis			✓	✓	✓	✓
	Cysteine and methionine metabolism				✓	✓	✓
	Tryptophan metabolism			✓	✓	✓	✓
	Tyrosine metabolism			✓			
	Phenylalanine metabolism						
	Glycine, serine, and threonine metabolism			✓	✓	✓	✓
	Phenylalanine, tyrosine, and tryptophan biosynthesis						
	Phenylalanine metabolism			✓		✓	
Nucleotide metabolism	Pyrimidine metabolism	✓	✓	✓	✓	✓	✓
	Purine metabolism						
Lipid metabolism	Glycerophospholipid metabolism		✓			✓	✓
	Linoleic acid metabolism					✓	✓
	Glycerolipid metabolism					✓	✓
	Cutin, suberine, and wax biosynthesis						
Biosynthesis of other secondary metabolites	Isoquinoline alkaloid biosynthesis			✓			
Biosynthesis of other secondary metabolites-unclassified					✓	✓	✓
Metabolism of cofactors and vitamins	Pantothenate and CoA biosynthesis			✓	✓		
Metabolism of other amino acids	Glutathione metabolism			✓	✓	✓	✓

*Em, embryo; Me, megagametophyte; SCR, seed coat rupture; RP, radicle protrusion. The symbol “✓” indicates significantly changed metabolic pathway between two time points.*

### Differential Metabolic Pathways Between DS-4 and NDS-1, and Between DS-4 and NDS-SCR

Carbohydrate metabolism and amino acid metabolism were the two major changed metabolic pathways between DS-4 and NDS-1, and between DS-4 and NDS-SCR (Table 3). There were more differential metabolic pathways in the embryo between DS-4 and NDS-1 than between DS-4 and NDS-SCR.

**TABLE 3:** The altered metabolic pathways in the embryos and megagametophytes between 4-weeks-incuabted primary physiological dormant seeds (DS-4) and 1-weeks-incubated non-primary physiological dormant seeds (NDS-1), between DS-4 and NDS at seed coat rupture stage (NDS-SCR).

**Metabolic pathways**		DS-4 vs. NDS-1		DS-4 vs. NDS-SCR	
		Em	Me	Em	Me
Carbohydrate metabolism	Pentose phosphate pathway	✓	✓	✓	✓
	C5-Branched dibasic acid metabolism				✓
	Fructose and mannose metabolism	✓	✓	✓	✓
	Amino sugar and nucleotide sugar metabolism	✓		✓	✓
	Pentose and glucuronate interconversions	✓	✓		✓
	Glycolysis/gluconeogenesis				
	Pyruvate metabolism				
	Glyoxylate and dicarboxylate metabolism	✓			
	Starch and sucrose metabolism	✓	✓		✓
	Galactose metabolism	✓	✓	✓	✓
	Citrate cycle (TCA cycle)	✓	✓	✓	
	Butanoate metabolism	✓	✓		✓
	Carbon fixation in photosynthetic organisms				
	Inositol phosphate metabolism	✓	✓	✓	✓
	Ascorbate and aldarate metabolism				✓
Amino acid metabolism	Alanine, aspartate, and glutamate metabolism	✓	✓	✓	✓
	Arginine and proline metabolism	✓	✓	✓	✓
	Arginine biosynthesis	✓	✓		✓
	Cysteine and methionine metabolism				✓
	Tryptophan metabolism			✓	
	Tyrosine metabolism	✓	✓		✓
	Phenylalanine metabolism				
	Glycine, serine, and threonine metabolism	✓	✓		✓
	Phenylalanine, tyrosine, and tryptophan biosynthesis		✓		
	Phenylalanine metabolism				
Nucleotide metabolism	Pyrimidine metabolism	✓			✓
	Purine metabolism	✓	✓		
Lipid metabolism	Glycerophospholipid metabolism				
	Linoleic acid metabolism		✓		
	Glycerolipid metabolism	✓			
	Cutin, suberine, and wax biosynthesis				
Biosynthesis of other secondary metabolites	Isoquinoline alkaloid biosynthesis	✓	✓	✓	✓
Biosynthesis of other secondary metabolites-unclassified		✓			
Metabolism of cofactors and vitamins	Pantothenate and CoA biosynthesis	✓			✓
Metabolism of other amino acids	Glutathione metabolism	✓	✓		✓

*Em, embryo; Me, megagametophyte.*

*The symbol “✓” indicates significantly changed metabolic pathway.*

## Discussion

### Alterations in Metabolic Profiles Between Embryo and Megagametophyte

In the present study, all measured metabolites were higher in the embryo than that in megagametophyte in both DS and NDS. It appears that both carbohydrate metabolism and amino acid metabolism are more predominant metabolic pathways in the embryo, but in megagametophyte amino acid metabolism becomes predominant. Similarly, several studies have demonstrated that there were different metabolic activities between the embryos and endosperm in *A. thaliana* (Penfield et al., 2006), rice (Galland et al., 2017), and wheat (Liu et al., 2018; Domergue et al., 2019). Xu et al. (2016) also reported that proteins with increased abundance in embryo were mainly associated with glycolysis and TCA cycle, cell growth, and division and protein synthesis during dormancy release of wild rice seeds. While, the abundances of these proteins in endosperms maintained steady-state level or decreased (Xu et al., 2016).

### Alterations in Metabolic Profiles Between DS-4 and NDS-1

It has been confirmed that ABA plays a critical role in the induction and expression of seed dormancy and the inhibition of seed germination in *A. thaliana* (Ali-Rachedi et al., 2004; Liu et al., 2013). Xia et al. (2018b) reported that the ABA level significantly accumulated in dormant sunflower seeds, but continued to decrease in non-dormant seeds after 24 h of imbibition. The 24 h imbibition was also the time when the metabolism of non-dormant seeds was clearly differentiated from that of dormant seeds. A rather similar pattern of change in the relative levels of ABA was found in the present study, with a great increase in the embryos of DS-4 and a rapid decline in the embryos of NDS-1. Similarly, there was a great variation in metabolism between DS-4 and NDS-1. It is therefore to hypothesize that ABA may affect the seed dormancy (or seed germination) process by its action on metabolic activities. Metabolic, transcriptomic, and proteomic analyses also showed that ABA regulated seed germination by inhibiting some metabolic processes in *A. thaliana* and sunflower (Chibani et al., 2006; Xia et al., 2018b).

It is generally accepted that glycolysis, fermentation, the TCA cycle, and PPP are activated to provide energy for metabolic activity during seed germination of several species, such as *A. thaliana* (Fait et al., 2006; Preston et al., 2009; Angelovici et al., 2011; Weitbrecht et al., 2011), rice (He and Yang, 2013), and *Cyclobalanopsis gilva* (Zaynab et al., 2018). It has been also suggested that energy is mainly provided by glycolysis and fermentation during an early stage of germination in rice (Yang et al., 2007; He et al., 2011; He and Yang, 2013), tree peony (*Paeonia ostii* ‘Feng Dan’) (Ren et al., 2018), and *C. gilva* (Zaynab et al., 2018), and TCA cycle is the main energy source at the late stage of germination. A decrease in TCA cycle activity would lead to germination inhibition in *A. thaliana* (Chibani et al., 2006; Angelovici et al., 2011), wheat (Das et al., 2017), and tree peony (*P. ostii* ‘Feng Dan’) (Ren et al., 2018). There were two larger metabolic switches at 1 and 4 weeks of incubation of DS, respectively. It has been reported that rapid uptake of water was occurred during the first 5 days of incubation of dry dormant Korean pine seeds (Song and Zhu, 2019). The increase in various metabolic activities in the embryos of DS during 1 week of incubation might be caused by rapid water uptake by dry DS. A transient but rapid increase in most metabolites occurred after 4 weeks of incubation when DS had already been imbibed, indicating that DS are far from a metabolically quiescent state when they were in favorable conditions. In contrast, the relative levels of various phosphorylated sugars, oligosaccharides, and most amino acids in the embryos of NDS substantially were decreased upon seed coat rupture. In addition, we found that the relative levels of a number of metabolites related to carbohydrate metabolism were significantly higher in the embryos of DS-4, especially those related to the PPP and TCA cycle, when compared to NDS-1. Although the relative contents of most metabolites were higher in the embryos of DS, it cannot be concluded whether the associated metabolic pathways are also active. Because we did not conduct further analysis in the present study, for example, genomics, transcriptomics, proteomics, and enzyme profiles analysis. One possibility could be that the accumulation of most metabolites related to carbohydrate metabolism may indicate a sudden halt in the TCA cycle. Korean pine seed primary physiological dormancy is thus maintained. Another possibility is that the high-efficiency operation of carbohydrate metabolism pathways may lead to the accumulation of related metabolites. It is therefore safe to hypothesize that carbohydrate metabolism, especially the PPP and TCA cycle, was more active in the embryos of 4-week-incubated DS as compared to 1-week-incubated NDS. Sugar enters to glycolysis and further the TCA cycle mainly through phosphorylation of sugars (Fait et al., 2006). A significant increase of numerous phosphorylated sugars and organic acids was involved in glycolysis, PPP and TCA cycle during incubation of DS may indicate an overaccumulation of reactive oxygen species (ROS), leading to seed dormancy. In contrast, the activities of the PPP and TCA cycle were attenuated most presumably to reduce the generation of ROS during incubation of NDS. The reduced PPP and TCA cycle may reflect a way to avoid energy waste and reserve consumption, preparing for the establishment of autotrophic growth of seedling after seed germination. Some results also suggest that the operation of the TCA cycle plays an important role in seed dormancy, but the attenuated TCA cycle is related to seed germination. For example, Xia et al. (2018a) detected a higher activity of enzymes related to the TCA cycle and glycolysis in imbibed-dormant sunflower seeds. Furthermore, sunflower seed dormancy can be released by cyanide (a respiratory inhibitor), implying the importance of respiratory activity in seed dormancy (Oracz et al., 2008, 2009). Non-targeted metabolomics analysis of poplar seeds also revealed that three TCA cycle intermediates (citrate, isocitrate, and succinate) were substantially accumulated during the periods of slow water uptake and then promptly decreased until radicle protrusion (Qu et al., 2019). Research conducted with non-dormant and dormant seeds of *Leymus chinensis* demonstrated that the proteins involved in the TCA cycle were decreased in non-dormant seeds (Hou et al., 2019).

The relative levels of amino acids in either embryos or megagametophytes were always significantly higher in NDS than that in DS during incubation. Most amino acids in the embryos of NDS were decreased substantially from 1 week to seed coat rupture, suggesting that amino acids might be utilized for protein biosynthesis required for seed germination. In contrast, only a few amino acids in embryos exhibited a slowly increasing trend during incubation of DS. Thus, we concluded that amino acid metabolism maybe involved in primary physiological dormancy in Korean pine seeds. Previous studies have shown that protein biosynthesis was inhibited in dormant seeds compared with non-dormant seeds in *Nicotiana plumbaginifolia* (Bove et al., 2005) and *A. thaliana* (Cadman et al., 2006; Carrera et al., 2008).

## Data Availability Statement

The raw data supporting the conclusions of this article will be made available by the authors, without undue reservation.

## Author Contributions

YS oversaw the project, produced the figures, and performed the statistical analyses. YS and XG wrote the manuscript. YW revised the manuscript. All authors contributed to the article and approved the submitted version.

## Conflict of Interest

The authors declare that the research was conducted in the absence of any commercial or financial relationships that could be construed as a potential conflict of interest.

## Publisher’s Note

All claims expressed in this article are solely those of the authors and do not necessarily represent those of their affiliated organizations, or those of the publisher, the editors and the reviewers. Any product that may be evaluated in this article, or claim that may be made by its manufacturer, is not guaranteed or endorsed by the publisher.

## Footnotes

1
https://www.metaboanalyst.ca/
